# Genetic Variations of African Swine Fever Virus: Major Challenges and Prospects

**DOI:** 10.3390/v16060913

**Published:** 2024-06-04

**Authors:** Shengmei Chen, Tao Wang, Rui Luo, Zhanhao Lu, Jing Lan, Yuan Sun, Qiang Fu, Hua-Ji Qiu

**Affiliations:** 1 College of Life Science and Engineering, Foshan University, Foshan 528231, China; 2 State Key Laboratory for Animal Disease Control and Prevention, National African Swine Fever Para-Reference Laboratory, National High Containment Facilities for Animal Diseases Control and Prevention, Harbin Veterinary Research Institute, Chinese Academy of Agricultural Sciences, Harbin 150069, China; 3 College of Animal Sciences, Yangtze University, Jingzhou 434023, China

**Keywords:** African swine fever, African swine fever virus, mutations, recombination, vaccines

## Abstract

African swine fever (ASF) is a contagious viral disease affecting pigs and wild boars. It typically presents as a hemorrhagic fever but can also manifest in various forms, ranging from acute to asymptomatic. ASF has spread extensively globally, significantly impacting the swine industry. The complex and highly variable character of the ASFV genome makes vaccine development and disease surveillance extremely difficult. The overall trend in ASFV evolution is towards decreased virulence and increased transmissibility. Factors such as gene mutation, viral recombination, and the strain-specificity of virulence-associated genes facilitate viral variations. This review deeply discusses the influence of these factors on viral immune evasion, pathogenicity, and the ensuing complexities encountered in vaccine development, disease detection, and surveillance. The ultimate goal of this review is to thoroughly explore the genetic evolution patterns and variation mechanisms of ASFV, providing a theoretical foundation for advancement in vaccine and diagnostic technologies.

## 1. Introduction

African swine fever (ASF) is a contagious viral disease affecting pigs and wild boars. It typically presents as a hemorrhagic fever but can also manifest in various forms, ranging from acute to asymptomatic [1]. ASF was first identified in Kenya in 1921 and has since spread across Africa, reaching Portugal in 1957 [2]. The genotype I strain was dominant during this period [3]. By the 1990s, most regions, except for Sardinia and some parts of Africa, had declared the successful eradication of ASF [4]. However, in 2007, genotype II ASFV emerged in Georgia and rapidly spread to Russia and other neighboring countries. In 2018, China suffered its first outbreak of ASF, characterized by high mortality rates and rapid transmission. As of the present, ASF has been reported in 80 countries worldwide, causing severe repercussions for the worldwide pig farming industry [5].

**Figure 1:**
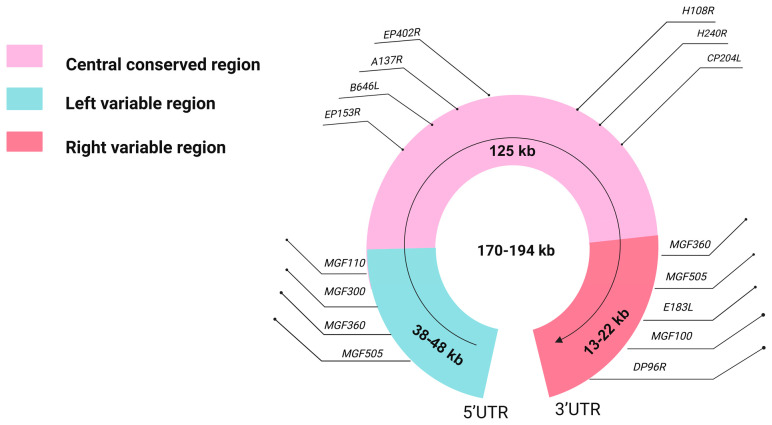
Structure of the ASFV genome. This figure shows the distribution of variable and conserved regions within the ASFV genome. Variable regions typically contain genes with significant sequence variability between different viral strains, whereas conserved regions comprise highly conserved gene sequences. This figure specifically annotates a series of genes that are relatively common in research. These genes are crucial for understanding the pathogenic mechanisms of ASFV and for developing vaccination strategies.

ASFV is a complex double-stranded DNA virus, with genome sizes varying from 170 to 194 kilobase pairs (kb) across different strains [6,7]. Structurally, the ASFV genome is organized into three distinct regions: the left variable region (LVR), the central conserved region (CCR), and the right variable region (RVR) [8,9]. The CCR remains relatively conserved across strains, while the variable regions contribute to genetic diversity (Figure 1). ASFV is classified into 25 genotypes based on variations in the terminal nucleotides of the *B646L* gene, which encodes the capsid protein p72 [10]. A recent study suggests that to improve the accuracy of ASFV classification and reduce redundancy, the current 25 *B646L*-based genotypes should be reduced to six [11]. Serotyping, traditionally based on the *EP402R* gene, which encodes CD2v, the hemadsorption-associated protein of ASFV, has identified nine serotypes, but latest methods using animal immune response mechanisms offer increased accuracy, integrating genetic and immunobiological markers like the hemadsorption inhibition (HADI) test and immunological testing on pigs inoculated with specific strains. This approach enhances precision in identifying virus lineages, providing a comprehensive framework for virus study and classification [12,13].

Over time, the focus on ASF has evolved from containment to the ambitious goal of total eradication, driven by advances in understanding the virus. New insights into the molecular epidemiology of ASF have highlighted three major concerns. Firstly, the general evolutionary trend of ASFV pathogenic strains is from high to low pathogenicity [14]. Secondly, extensive recombination among viral strains has been discovered and substantiated significantly [15]. Thirdly, the virulence of ASFV strains is characterized by strain-specificity. A range of challenging issues arises from these revelations [16,17]. First and foremost, the gradual attenuation of virulence, juxtaposed with enhanced transmissibility, effectively cloaks the presence of the virus, making eradication efforts more difficult. Moreover, recombination has put the evolutionary mutations of ASFV on the fast track to additional biosecurity and disease prevention challenges. Last but not least, the strain-specificity of the virulence-associated genes (VAGs) in ASFV, resulting from its complex genomic structure, is a major impediment to vaccine research and development and a formidable obstacle to effective ASF control [18].

## 2. Two Major Ways of Genetic Variations in ASFV

### 2.1. Genetic Mutation

Genetic variations in ASFV are driven primarily through mutation and recombination, with mutations involving single-nucleotide polymorphism (SNPs), insertions, and deletions [19]. These genetic mutations, particularly insertions and deletions within gene sequences, serve not only as the foundation for genetic diversity within ASFV populations but also as the primary driving force behind its genetic variations [20]. Based on existing biotechnological tools, we can predict the impact of variations in single nucleotides on viral functional proteins with relative accuracy, and the changes caused by such SNPs tend to be of minor impact. In contrast, many deletions and insertions in the viral genome may severely affect the protein’s function, leading to the loss of the entire functional protein, with major implications for viral adaptation and evolution [21]. Research has shown that in contrast to typical DNA repair pathways, both the DNA polymerase (PolX) and the ligase (LIG) in the ASFV DNA repair pathway exhibit significantly low fidelity [22]. This is a major contributing factor to the frequent and extensive insertions and deletions observed within ASFV genomic segments. Although the precise molecular mechanisms underlying the low fidelity of ASFV LIG and ASFV PolX remain unclear, recent studies have provided insight into unique structural features of ASFV LIG, such as the N-terminal domain (NTD) and specific active site residues (including Asn153, Leu211, Leu402, and Gln403) [23]. These features likely facilitate erroneous pairings during DNA repair catalysis. Similarly, the low fidelity of ASFV PolX can primarily be attributed to its lack of proofreading 3′-5′ exonuclease activity and the absence of crucial DNA-binding domains, including the thumb and 8-kilodalton (KD) domains. Additionally, a unique 5′-phosphate binding pocket in ASFV PolX promotes the misinsertion of dGTP, thus increasing mutation rates [24,25,26]. These structural and functional characteristics lead these enzymes to frequently introduce point mutations, insertions, deletions, and replication slippage during DNA replication and repair processes, potentially resulting in coding errors, frame-shift mutations, and sequence duplications. These mutations enhance the virus’s genetic diversity and its ability to resist host immune responses and therapeutic interventions.

### 2.2. Genetic Recombination

Genetic recombination is another critical way of genetic variation. The definition of recombination in virology lacks consistency due to its complexity. Some researchers posit that when a host cell is infected by two or more strains of ASFV from different origins or possessing distinct genetic traits, there is a certain probability of genetic recombination occurring during the nucleic acid replication processes [27,28]. However, capturing and observing this process is challenging. Traditionally, the discovery of viral recombination has predominantly focused on RNA viruses, with recombination considered a common characteristic among them. This is because the replication mechanism of RNA viruses is more prone to errors, thereby increasing the opportunities for genetic variation and recombination. Additionally, the high genetic diversity in RNA viruses makes them more adaptable to environmental changes through mechanisms like recombination, enabling them to evade the host’s immune response more effectively [29]. The discovery of recombination in ASFV provides substantial theoretical support for the study of recombination in DNA viruses, suggesting that DNA viruses, like RNA viruses, can exhibit high rates of recombination. This discovery has greatly advanced the field of viral recombination research and has provided valuable insights into the mechanisms underlying viral gene exchange [30,31]. On farms, as ASFV spreads among different individuals of the same species, genetic material from different strains may mix, facilitating genetic exchange through recombination events. This process can lead to the emergence of recombinant strains, which may have altered biological properties. In natural habitats, ASFV circulates between individuals of different species, including domestic pigs, wild boar, warthogs, and soft ticks. Given the variations in host species, the virus must constantly adapt to different hosts, resulting in a co-evolutionary dynamic between the virus and its hosts. Particularly noteworthy is the symbiotic relationship ASFV forms with soft ticks [32,33,34]. This adaptability enables the virus to continuously evolve and recombine, adapting to the life cycle of soft ticks and ultimately optimizing its ability to spread efficiently. Recombination may lead to the acquisition of new VAGs, enhancing virulence. However, it can also sometimes result in decreased virulence. This reduction can benefit the viral strain by promoting symbiosis with the host and potentially increasing transmission rates. Additionally, recombination could lead to alterations in antigenic phenotypes, reducing cross-protection. Initially, the hypothesis that recombination could occur between different ASFV genotypes was speculative, based on early research, and lacked concrete evidence. It was not until 2023 that this hypothesis was confirmed when Bu et al. reported the discovery of recombinant viruses between ASFV genotypes I and II, providing conclusive evidence that recombination between different ASFV genotypes is possible [15]. The recombination of the ASFV has pushed its evolution into the fast lane, making it increasingly adaptable and more potent. Therefore, it is not only a priority but a necessity to increase our focus on studying ASFV genetic variations. In this fierce race against the rapid mutation of ASFV, strengthening research on ASFV recombination and improving prevention and control strategies are our best bets to stay one step ahead.

## 3. Main Trends in the Genetic Evolution of ASFV

### 3.1. Reduced Virulence and Increased Transmission

Mutations in the genomic sequence of ASFV predominantly appear as extensive insertion and deletion events, strikingly including large deletions that seemingly do not disrupt the virus’s life cycle [35]. This peculiarity could be ascribed to the compensatory mechanisms inherent in the large ASFV genome, which may mitigate the effects of reducing protein functionality. Out of the many insertion and deletion events, the genes that had the most significant impact were multigene families (*MGFs*) and *EP402R* [36,37,38]. During the 2020 incursion into Germany, ASFV unexpectedly diverged into five distinctly different lineages, each exhibiting at least 10 unique variants characterized by previously unidentified high-impact mutations. A comparison of the genetic evolution of the German ASFV variants with the ASFV strain Georgian 2007/1 revealed 13 distinct insertions and deletions, primarily concentrated in the *MGF360* and *MGF505* regions [39]. Further investigations revealed a strong positive correlation between the genetic diversity of ASFV and the amount of insertions and deletions events in the *MGF* genes [19]. Moreover, these genetic mutations display significant sequence variation across different ASFV strains [40]. The widespread occurrence of insertion and deletion events within the genome has led to an evolutionary adaptation in the epidemic strains, resulting in a reduction in lethality combined with an increase in infectivity. This transformation made the transmission of ASFV more covert, significantly obstructing efforts aimed at its eradication.

Research has documented that ASFV strains, isolated from wild pigs in Russia, Lithuania, and Estonia exhibit an incubation period of up to 21 days when inoculated into specific-pathogen-free (SPF) pigs. Remarkably, these infected pigs either showed no symptoms or did not succumb to the disease, suggesting that widespread transmission of ASFV might reduce virulence [41,42]. In another study, virulence challenge experiments were conducted on 15 ASFV strains isolated from various regions of Russia between 2013 and 2018. The results indicated that eight of these strains exhibited high virulence characteristics, while seven showed moderate to low virulence. Among the eight high virulence strains, three exhibited minimal differences in mortality days across varying doses, all succumbing within 1 to 14 days. In contrast, five of the moderate- to low-virulence strains displayed variations in survival times due to differences in doses, with all deaths occurring after 14 days at lower dosages, categorizing them as moderate- to low-virulence strains. Despite potential discrepancies due to sampling methods, the detection of a significant number of moderate- to low-virulence strains provides evidence of a trend toward decreased virulence [43].

Further evidence from recent Chinese studies supports this trend, where gene deletions and protein expression defects have lowered ASFV virulence. A study from June to December 2020 across seven Chinese provinces discovered that 11 out of 22 ASFV isolates had four distinct mutation or deletion types in the *EP402R* gene. Notably, hemadsorption (HAD) test results for these isolates were all negative, particularly in the case of the HLJ/HRB1/20 strain, which has a deletion of 25 nucleotides at positions 43–67 in the *EP402R* gene. This deletion inhibits complete CD2v translation, and subsequent tests confirmed the reduced virulence of HLJ/HRB1/20 [44]. Comparative challenge experiments between the YNFN202103 strain and the virulent strain GZ2018/01/2 showed that YNFN202103 had a 20% lower mortality rate, prolonged onset and survival times, and lower viremia levels despite significantly higher challenge doses. Despite similar overall viral copy numbers, YNFN202103 demonstrated reduced virulence and increased infectivity [45]. In summary, numerous attenuated ASFV strains have emerged across China over the past two years, a phenomenon likely resulting from the natural attenuation of ASFV virulence due to its prevalence in China’s extensive swine population.

This situation has been observed in other countries besides China, where an evolutionary trend of ASFV from high to low pathogenicity has been observed. For instance, after its introduction in Eastern Europe in 2007, the high-virulence strain Georgia-2007/1 was isolated in Georgia. Towards the end of 2011, a low-virulence strain, Dilijan2011IMB, was identified in Armenia [46]. As the infection persisted in this region, the clinical presentation of ASF gradually became atypical. Specifically, between 2018 and 2020, comprehensive animal experiments and pathological analyses were conducted on 55 ASFV strains isolated from 14 farms in the Caucasus region. This investigation identified three strains with moderate pathology and low mortality rates, supporting the trend of evolving towards reduced pathogenicity [47]. Similarly, Western Europe supports the conclusion that ASFV evolves from high virulence to low virulence. The high-virulence strain Lisboa60 (L60) and the low-virulence strain NH/P68 (NHV), having both emerged in Portugal but eight years apart, share a genomic homology of 99.65%. Compared with the L60 strain, the NHV strain incorporates additional genes such as *MGF100-1R*, *MGF110-4L*, *MGF110-5L*, and *MGF110-9L* into the ASFV genome, alongside an insertion of 4458 nucleotides between *MGF110-2L* and *MGF110-13L*. The reduced virulence of NHV may be due to changes in the biology of ASFV caused by mutations in these genes, which in turn affects its virulence [48].

The lack of HAD ability has always been a key factor in reducing the virulence of ASFV. Researchers have analyzed 71 whole-genome sequences of ASFV collected during a 40-year epidemic on Sardinia, revealing that nearly all virus strains isolated after 1990 exhibit deletions in the *B602L* and *EP402R* genes [49]. In contrast, historical strains isolated before 1990 do not show these deletions. As an isolated system, Sardinia provides an ideal setting for studying the natural variations of ASFV in a relatively independent environment, thereby reflecting the virus’s natural evolutionary trends more accurately. Studies indicate that the deletion of the *EP402R* gene is quite common in Sardinia. Given that the *EP402R* gene is described as being associated with virulence in most references, these findings allow for us to speculate boldly that the evolutionary trend of ASFV is toward reducing HAD capability, thereby decreasing virulence [50,51].

**Figure 2:**
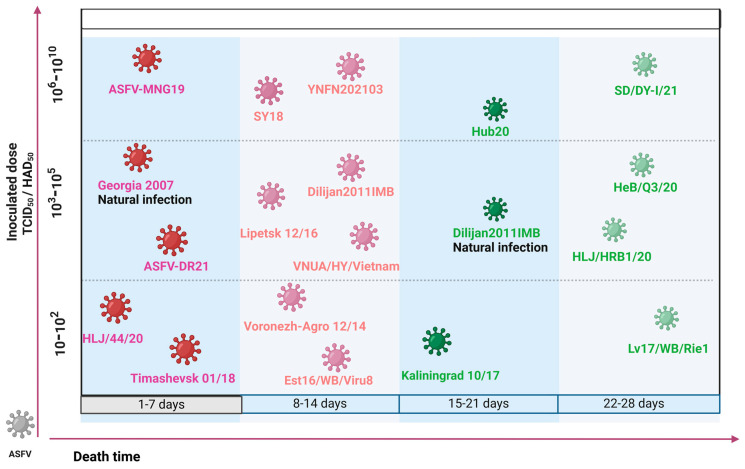
Diagram of the pathogenicity of different ASFV strains. In this illustration, strains marked in green represent low-virulence strains, while those marked in red indicate high-virulence strains. The depicted experimental outcomes are primarily sourced from pathogenicity tests in SPF pigs. Additional information can be found in Refs. [42,44,51,58,59,60,61,62,63].

However, not all regional ASFV strains exhibit a trend toward reduced virulence. Epidemiological studies in South Korea from 2019 to 2021 indicated no significant changes in the pathogenicity of circulating strains, confirmed by animal studies. In another study in South Korea, researchers conducted virulence tests on two strains isolated between 2022 and January 2023, suspected to have decreased pathogenicity. However, the experimental outcomes revealed that all inoculated pigs succumbed to the infection within 8 to 10 days, displaying acute pathological symptoms [52]. These findings suggest that, throughout the study period, the pathogenicity among South Korean ASFV isolates remained consistent, underscoring the stable virulence profiles of ASFV strains in the region. Although there are regional differences in the study outcomes of ASFV pathogenicity, these discrepancies can be attributed to factors such as the extent of disease spread, modes of transmission, and the selection of isolates [53]. Nonetheless, driven by the forces of natural selection, the evolutionary trend of ASFV persistently gravitates towards reduced pathogenicity and heightened transmissibility, echoing the law of genetic evolution of organisms (Figure 2). 

### 3.2. Evolutionary Drivers of Reduced Virulence

The most likely explanation for the reduced virulence of the above ASFV is due to the evolutionary nature of viruses. Under the natural selection phenomenon, the virus’s genome undergoes continuous changes, consistently evolving in a direction that enhances its survival and adaptation. Studies on viral evolution have demonstrated that if viruses are given sufficient time, these genomic changes almost inevitably lead to decreased pathogenicity [54]. Genetic variations of ASFV are an extremely complex process, which may result in attenuated virulence due to mutations in VAGs on the one hand and the evolution of more virulent strains due to genetic recombination between strains on the other hand. Driven by host immune pressures, ASFV strains generally show a trend toward decreased virulence. This trend results from the selective pressures inherent in the evolutionary process [55,56]. Although, in most cases, genetic deletions appear to have had a detrimental effect on viral adaptation, those deleted viral strains that have been able to persist and remain stably fixed in viral populations suggest that these mutations confer some adaptive advantages. These advantages may include but are not limited to better evasion in the host immune response or enhanced transmission under specific environmental conditions, which may be favored by natural selection and retained in the evolutionary process. As ongoing research continues to isolate and identify more ASFV strains, an increasing number of attenuated strains with potential vaccine value are emerging worldwide [57]. However, mutations may also lead to the development of strains that possess enhanced immune evasion and more covert transmission advantages. This situation presents a significant challenge for the prevention and control of ASF outbreaks, highlighting the complexities involved in managing the disease amid ongoing viral evolution.

## 4. Genetic Recombination of ASFV

### 4.1. Impact of Recombination on ASFV

**Figure 3:**
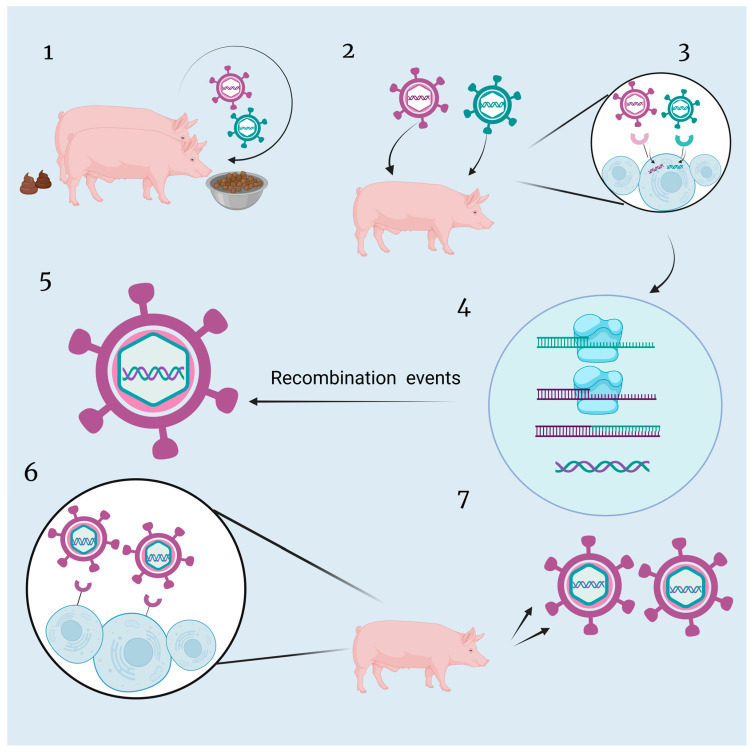
The hypothetical framework of recombination pathway of ASFV. (1) Two independent hosts transmit their respective ASFV strains through horizontal transmission. (2) Two distinct ASFV strains simultaneously infect a single host pig. (3) Both ASFV strains synergistically invade and infect the same target host cell. (4) DNA fragments from the two ASFV strains undergo simultaneous replication and genetic material exchange within the host cell. (5) Translation of new proteins based on new genetic material and successful assembly into recombinant viruses. (6) The recombinant ASFV particles continuously replicate and assemble within the host, forming mature virions. (7) The recombinant strain with novel genetic characteristics demonstrates increased infectivity and viability, further spreading throughout the host population.

In addition to genome deletions and insertions, another highly mutable process observed in ASFV is the phenomenon of viral recombination (Figure 3). Viral recombination is a more rapid way of changing the structure of the viral genome. At first, it was believed that RNA viruses were more likely to undergo recombination of their genes, but DNA viruses, such as ASFV, were considered to undergo only a limited number of recombination events. However, as more and more evidence of recombination has been found in ASFV, recombination events in ASFV as a DNA virus have garnered widespread attention. Recombination is associated with various functions, such as expanding the host range of the virus, increased virulence, evasion of host immunity, and drug resistance [64,65,66,67]. However, from the viral perspective, the recombination was not easy and required the breaking down of barriers. Initially, two distinct ASFV strains must infect the same host and coincidentally invade the same host cell. Then, similar DNA fragments from the two strains undergo replication and engage in fragment exchange simultaneously. The resulting recombinant virus retains the capabilities for infection, invasion, replication, and assembly, becoming the dominant strain, surpassing the survival and replication efficiency of other viral strains. Ultimately, this strain reaches a sufficient level of infection to be successfully isolated by scientists. Owing to the complexity of viral recombination mechanisms, several questions about recombination among DNA viruses remain unanswered, in particular, whether their propensity to recombine correlates with their genomic susceptibility to deletions and insertions [68].

### 4.2. Case Studies on ASFV Genetic Recombination

Previously, the Georgia-2007/1 isolate was classified as a genotype II ASFV strain based on partial sequencing of the *B646L* and *B602L* genes, along with complete sequencing of the *CP204L* gene. To further understand the genetic relationships between Georgia-2007/1 and other ASFV isolates, researchers performed a comparative analysis of the amino acid sequences encoded by 125 conserved ORFs. This analysis revealed that Georgia-2007/1 has a closer phylogenetic relationship with the Mkuzi/1979 strain (genotype I) but also displayed significant differences in certain ORFs, indicating an unusual evolutionary pattern in some genes. When comparing to the ASFV strain Mkuzi/1979, it was found that most genes in Georgia-2007/1 showed high homology, yet there were genes, such as *EP153R*, that had higher homology with other isolates and lower homology with Mkuzi/1979. These findings indirectly suggest that Georgia-2007/1 may have undergone recombination events [68].

In 2023, three deadly ASFV strains (JS/LG/21, HeN/123014/22, and IM/DQDM/22) were identified in China, resulting from a recombination of genotypes I and II. While the virus samples were initially identified as genotype I, all HAD test results were negative. Using a systematic approach, researchers divided the recombinant virus genome into 20 fragments, revealing 10 from genotype I (SD/DY/I/21) and 10 from genotype II (HLJ/18). Animal tests indicated that JS/LG/21 exhibited high lethality. Even the live attenuated vaccine (LAV) HLJ/18/7GD, developed based on genotype II, failed to provide immune protection against this recombinant virus [15]. This suggests that the antigenic alterations in recombinant strains and the increased viral escape ability present new challenges for ASF outbreak prevention and control. 

### 4.3. Challenges of Virus Recombination for Controlling ASF

Among the recombinant strains that have been identified, those resulting from recombination between genotype I and genotype II of ASFV have shown increased virulence. The existing candidate vaccine strain, ASFV-HLJ/18-7GD, lacks the capability for cross-protection against these recombinant strains, presenting new challenges for the control and prevention of ASF [15]. This situation necessitates further research and monitoring of recombinant viruses’ transmission and pathogenic mechanisms. The antigenic diversity exhibited by recombinant strains poses a significant obstacle to developing LAV. In the practical application of RNA virus vaccines, many cases of recombination between wild and vaccinated strains have led to the emergence of new strains. This situation presents two significant challenges: firstly, the new recombinant strains may carry some characteristics of the wild strains or other characteristics of the vaccinated strains, resulting in the expression of antigenic properties different from those of the original strains. This complicates the distinction between wild and vaccine strains, as they may share similar antigenic characteristics, significantly impacting outbreak decontamination efforts. A prominent example of this is porcine reproductive and respiratory syndrome (PRRS) in China. Secondly, recombination between vaccine and wild strains could potentially enhance virulence. Although rare, this could lead to vaccinated individuals experiencing more severe pathological symptoms than expected or the spread of new, more harmful virus strains within swine herd [69]. In summary, the discovery of recombinant ASFV strains has reshaped our understanding and introduced new challenges to vaccine development.

## 5. The Strain-Specificity of the ASFV VAGs

The genome of the ASFV is currently recognized as one of the larger genomes among known viruses, capable of encoding hundreds of proteins [70]. Its complex composition, along with the interactions between its various genes and the translation of VAGs, has resulted in the emergence of numerous unpredictable phenotypic expressions. Among these, the most typical is known as the strain-specificity of VAGs, referring to as the unique expression patterns of certain genes in specific viral strains. This phenomenon was initially observed through significant differences between particular strains [71]. The emergence of such strain-specificity of VAGs poses significant challenges to vaccine development, particularly in ensuring vaccine safety.

### 5.1. Strain-Specificity of the EP402R

Among the structural proteins of ASFV, CD2v, encoded by the *EP402R*, is a critical protein for inducing HAD, playing a key role in the interaction between ASFV and the host immune system, and is also the primary antigenic protein of the virus. Typically, deficiencies in the CD2v protein impair the HAD ability of ASFV, reducing virulence. For example, the low-virulence ASFV strain HUB20 from China demonstrates insufficient expression of CD2v, resulting in reduced pathogenicity [72]. This implies that deletions in the genome, which alter protein structures, may result in the loss of crucial functions, thereby reducing ASFV virulence. Such a process aligns with the evolutionary trend of viruses towards reduced virulence and increased transmissibility. However, anomalies have been discovered against this broad background of evolution. Despite the loss of the functions of CD2v, ASFV strains such as Malawi-Lil-20/1 and Georgia 2010 still exhibit high pathogenicity similar to their parent viruses [16,73]. These anomalies may be related to the complex genomic structure of ASFV and its compensatory mechanisms after protein deletions [74]. This deviation illustrates the complex interplay of genetic variations and their unpredictable impact on ASFV pathogenicity, challenging established notions of viral evolution and protein function. Additionally, recent international research has reported that the ASFV-K49 strain from Congo, after deletion of *EP402R*, did not exhibit changes in virulence or replication capabilities *in vitro* within primary porcine alveolar macrophages (PAMs) [36]. However, *in vivo* experiments showed that animals infected with K49∆CD2v had limited ability to replicate and spread the virus, suggesting that different environmental factors may affect the survival and replication ways of ASFV. The studies discussed highlight variations in the expression of CD2v, one of the typical VAGs, among different ASFV strains. The specific mechanisms underlying these variations remain to be unraveled, attributable to the complexity and effect of various factors. An important factor likely contributing to this phenomenon is the genetic background or genomic structure of ASFV strains [75,76,77].

### 5.2. Strain-Specificity of the MGF Genes

*MGFs*, initially identified as genes encoding important non-structural proteins, were recognized as VAGs of ASFV in 2015. Mutations in *MGFs* generally lead to decreased pathogenicity of ASFV. For example, significant deletions in *MGF360* and *MGF110* of the ASFV genome are crucial factors in the evolutionary process, leading to the low-virulence strain Estonia 2014 from the highly virulent strain Georgia 2007/1 within the same genotype [78,79]. However, contrary to the prevalent notions that deletions in specific segments of the *MGF* genes could substantially reduce the virulence of ASFV, extensive research has demonstrated that the expression of the *MGF* genes varies significantly among different strains. For instance, a mutant generated by deleting *MGF505-7R* from the virulent CN/GS/2018 strain (referred to as ASFV-D7R) was used to infect six 3-week-old SPF pigs at a dose of 10 HAD_50_, and all pigs survived. In contrast, pathogenicity in pigs is maintained when *MGF505-7R* is deleted in the virulent HLJ/18 strain [79,80]. These studies imply that the influence of *MGFs* on the pathogenicity of the ASFV is strain-specific, with variability possibly resulting from genetic differences between ASFV isolates or changes in the expression of *MGFs* and neighboring genes during the generation of ASFV variants lacking *MGFs*. Such hypotheses necessitate further experimental validation.

### 5.3. Strain-Specificity of Other VAGs

When considering strain-specificity, the *DP148R* gene in ASFV is a critical example. Deletion of the *DP148R* gene in the ASFV strain Benin-97/1 significantly diminishes its replication capability and viral virulence in PAMs [81]. Conversely, the HLJ/18 strain retains its lethality even in the absence of the *DP148R* gene. Similarly, removal of the *DP148R* in the Georgia 2007/1 isolate, either alone or in combination with the *K145R* gene, did not reduce the ability of the virus to replicate in PAMs, and it still exhibited typical acute symptoms of ASFV [82]. The reason for the different outcomes may be attributed to differences in the presence or sequence variations of the genes encoding other VAGs in their genomes. While *DP148R* exhibits a high sequence similarity between the Benin 97/1 and Georgia 2007/1 strains, they may interact differently with other genes and genomic environmental factors, leading to distinct phenotypic expressions. These differences could affect the function of *DP148R*, as well as the growth and pathogenicity of the virus.

### 5.4. Implications of Strain-Specificity of VAGs

The aforementioned contrasting studies suggest that the variations in viral phenotypes resulting from the deletion of VAGs in different ASFV isolates may differ from one virus strain to another (Table 1). ASFV has a large genome, which may be a significant factor contributing to this phenomenon. When certain proteins are deleted in a viral strain, complex interactions between the remaining proteins and genes are triggered, orchestrating a compensatory response [83]. This mechanism, similar to a complex self-regulation process, ensures the sustained operational integrity of the virus despite genetic deficits. The reasons behind these strain-specific differences remain unclear, but it cannot be ruled out that it is influenced by the individual characteristics of the experimental animals and by differences in experimental conditions. The strain-specificity of VAGs is a significant factor limiting the development of ASF vaccines, particularly for gene-deleted LAVs. LAVs may undergo virulence reversion due to intrinsic variations or differences in host environments. Although cases of virulence reversion in vaccine strains have not been observed to date, it remains an issue that cannot be overlooked in vaccine development. The molecular mechanisms underlying this phenomenon are not fully understood, which calls for deeper research to unravel the complexities involved.

## 6. Control Strategies

**Figure 4:**
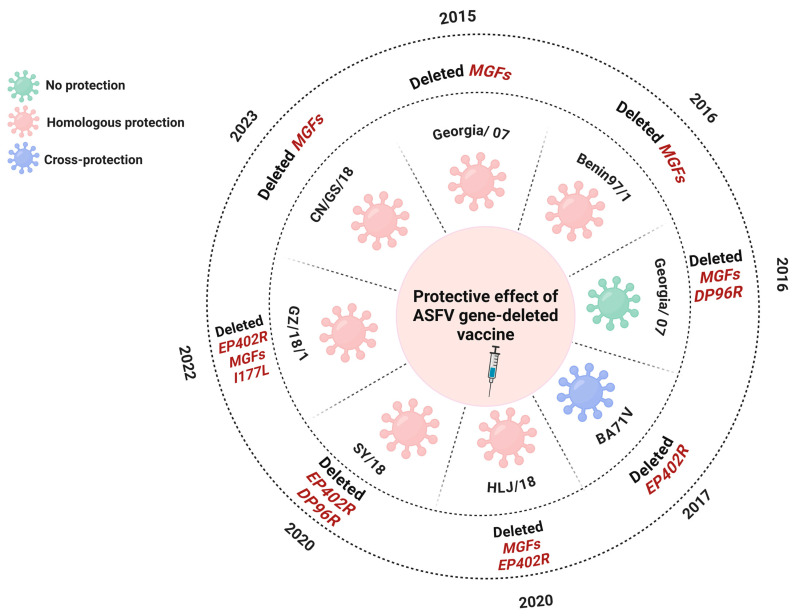
Diagram of differential protection of the gene-deleted ASFV LAVs. This chart illustrates the protection of various gene-deleted ASFV LAVs developed over the past years. Distinct colors signify different levels of immunoprotection: green indicates no protection, pink represents homologous protection, and blue signifies cross-protection. Deletions of specified genes, such as *EP402R*, *MGFs*, and others, are annotated alongside the year of report.

The quest for a practical vaccine against ASFV has been stymied by its intrinsic challenges, notably its high rate of mutation, its susceptibility to recombination, and the presence of strain-specific VAGs. These elements significantly obstruct the fulfillment of vaccine development’s safety and efficacy criteria, with the mutation and recombination capabilities undermining efficacy and VAG specificity posing serious safety concerns. Given that no neutralizing antibodies have been identified for ASF to date, research and development efforts for vaccines are increasingly focused on cellular immunity, with LAV considered the most promising type of vaccine for ASF. Current endeavors in LAV development predominantly revolve around generating candidate vaccine strains through targeted deletion of single or multiple VAGs. Attenuation strategies typically involve gene deletions with *MGFs* or genes related to HAD [84,85,86]. Researchers have long been working on the development of LAVs with mixed results. Some vaccines offer effective homologous protection against the same strain or cross-protection against different strains, while others have fallen short of expectations (Figure 4). Most of the results of LAV research are limited to the homologous protection provided by vaccines and are less effective in terms of cross-protection [87,88].

The complex and unpredictable nature of the ASFV genome raises concerns about the potential emergence of strain-specific responses in future vaccines, posing significant safety risks. Accumulating data indicate that deleting six *MGF* genes (*MGF505-1R*, *MGF360-12L*, *MGF360-13L*, *MGF360-14L*, *MGF505-2R*, and *MGF505-3R*) from the highly virulent ASFV-G, the mutants can fully protect against the deadly challenge posed by the parent virus, with only a minority of pigs showing brief episodes of fever [8]. Furthermore, the mutant obtained by deleting these six genes in HLJ/18 was completely attenuated in virulence and induced complete protection against the parental potent virus. However, it has been reported that the vaccine candidates (HLJ/18-6GD) reverted to virulence after six back-passages, and it remains unclear how this reversal occurred [89,90]. As the understanding of ASFV has grown, the LAVs that have developed have become more effective and stable in providing protection. In 2020, a vaccine candidate against ASFV was generated by deleting seven genes from the ASFV strain HLJ/18. The modified strain, known as HLJ/18-7GD, was completely attenuated in pigs, showing no signs of virulence reversion. Moreover, it provides complete immune protection against virulent strains [91,92]. However, recent studies have confirmed the limited cross-protection of HLJ/18-7GD against the recombinants composed of genotype I and II ASFV strains [15]. This highlights the potential impact of the high mutability and recombination-prone nature of ASFV genes, which may result in poor cross-immunoprotection, significantly affecting vaccine efficacy.

Vaccine development requires consideration of both safety and efficacy. Given the numerous studies expressing concerns about the inadequate safety profile of LAV, many scholars have redirected their attention toward the co-immunization approach [93,94]. This strategy combines LAVs with subunit vaccines to leverage the benefits of both, aiming to optimize vaccine efficacy. This method effectively addresses the issues of insufficient cross-protection offered by LAVs and the limited efficacy of subunit vaccines, presenting a promising avenue for achieving comprehensive cross-immune protection against the highly variable ASFV strains [95,96]. It is worth noting that while co-immunization is a promising strategy, it remains unclear which ASFV proteins are effective immunogens due to the vast and complex ASFV genome. The identification of effective immunogens is crucial for coping with highly variable ASFV strains [97,98].

In conclusion, the path forward in the evolutionary study of ASF remains arduous. On the one hand, a deeper understanding of the functions and interactions of ASFV proteins is needed to provide essential theoretical support for vaccine development. This should take into account the diverse genotypes of ASFV and employ varied strategies to enhance immune efficacy [99,100]. On the other hand, it is important to recognize that exposure of pigs to viruses does not always lead to disease, as a specific threshold of infection is necessary for disease manifestation. We must adopt a systematic and multi-pronged approach that includes comprehensive, consistent management measures for prevention and control. This strategy aims to effectively reduce the viral load, lightening the burden, while simultaneously fortifying the herd’s immune defenses, akin to strengthening a shield, to diminish the presence of the virus on the farm and improve the immunity of pigs [101,102,103]. Ultimately, ASF can be eradicated through an effective ASF prevention strategy that focuses on both technology and management.

## 7. Conclusions and Prospects

The highly efficient mutation of ASFV is like an accelerating engine, providing a strong impetus for genetic evolution, allowing for the virus to evolve towards low virulence and high infectivity by natural selection [104,105,106]. Under this major evolutionary trend, we also found some special phenomena, including the discovery of strain-specificity of VAGs and recombinant ASFV strains with different genotypes. These findings enriched the diversity of ASFV and presented new challenges in the realms of ASF outbreak prevention, control, and vaccine development.

The diminished virulence and enhanced transmissibility of viruses render the infection process more covert, broader in scope, and quicker in spread, posing a serious challenge to our outbreak prevention and control work and making it necessary for us to increase the cost of strengthening quarantine and disinfection measures, as well as the surveillance of infections on a large scale.

Regarding the strain-specificity of VAGs, this issue introduces an additional obstacle to vaccine development about vaccine safety. The current literature suggests that deletion of VAGs is the most promising approach for the development of an effective ASFV vaccine. Yet, the mere absence of VAGs does not equate to safety since changes in ASFV virulence are not caused by the lack of a single virulence gene but result from the interplay of multiple genes or the genetic background and genomic structure differences among various ASFV strains. Hence, these factors should be fully considered in the development of LAV, and more research is needed on the interactions between the different genes of ASFV. Additionally, the ability of LAV to cross-protect against emerging recombinant strains of the virus is another challenge we need to face. The discovery of recombinant strains with different genotypes accelerates the mutation of ASFV genes and also leads to the lack of cross-immunoprotection effect of the vaccine, which significantly affects the vaccine’s efficacy.

Moreover, recombinant strains add a significant dimension to the evolution of ASFV. On one hand, recombinant strains composed of genotypes I and II exhibit unique advantages in virulence, which falls between that of traditional highly virulent and less virulent strains. Due to this characteristic, it is speculated that recombinant strains will become the predominant strains of ASF prevalence worldwide. For a virus to become epidemic, it must replicate within hosts and spread effectively between individuals. Yet, one virus with high virulence can lead to rapid host mortality, which in turn can restrict the chances of its spread. Conversely, a virus with pathogenicity that is too weak might be defeated in its battle against the immune system. Therefore, a virus strain that is not lethal but capable enough to spread is more in line with microbial evolutionary trends, becoming prevalent within a region. This explains why “intermediate” recombinant virus strains are predicted to become the dominant strains for a period in the future. Nevertheless, virus evolution tends to progress towards reduced virulence, with recombinant strains only temporarily prevailing. The ultimate trend in virus development is towards a better coexistence with the host, a milestone that still requires a long journey ahead. On the other hand, the emergence of recombinant ASFV strains poses new challenges for vaccine development. It is essential not only to guard against the potential for enhanced virulence resulting from recombination between vaccine and wild strains but also to be vigilant for possible insufficient cross-immune protection between vaccine strains and recombinant strains.

Vaccines are a crucial tool for eradicating or controlling diseases in humans and animals, as evidenced by the eradication of smallpox. However, vaccine development is slow compared with the disease’s rapid emergence and mutation. While strategies to attenuate ASFV hold promise for effective vaccines in commercial pig populations, ASFV’s genetic diversity presents a significant challenge. Thus, relying solely on vaccines is insufficient for disease control. Before an effective vaccine against ASF is developed, our strategies are to create an environment unfavorable to the virus and enhance the health management of pigs. This includes maintaining dryness and cleanliness in pig farms to reduce virus survivability, as well as preserving the integrity of the pig skin and mucous membranes and boosting the immune system. In this way, even if a low-amount virus is ingested, it does not necessarily lead to a disease. If the quantity of the virus is insufficient to trigger an infection or if a pig possesses a strong enough immune system to eliminate the virus quickly, then the likelihood of a disease significantly decreases. By implementing a comprehensive biosecurity strategy, we aim to minimize viral impacts and enhance the natural defense of pigs.

## Figures and Tables

**Table 1:** The Strain-Specificity of ASFV VAGs.

Genes	Strain	Injected Dose	Genotype	Effect of Gene Deletion on Virulence	References
*EP402R*	HUB20	10^3^ TCID_50_	I	Decreased	[69]
*EP402R*	Georgia 2010	10^3^ TCID_50_	II	Unchanged	[73]
*EP402R*	Malawi-Lil-20/1	10^3^ TCID_50_	II	Unchanged	[73]
*MGF360*	Georgia 2007/1	/	II	Decreased	[77]
*MGF110*	Georgia 2007/1	/	II	Decreased	[77]
*MGF505-7R*	CN/GS/2018	10 HAD_50_	II	Decreased	[78]
*MGF505-7R*	HLJ/18	10^2^ HAD_50_	II	Unchanged	[79]
*DP148R*	Benin-97/1	10^3^ HAD_50_	I	Decreased	[80]
*DP148R*	HLJ/18	10^3^ HAD_50_	II	Unchanged	[62]
*DP148R*	Georgia 2007/1	10^3^ HAD_50_	II	Unchanged	[81]

## Data Availability

Not applicable.
